# A Statistical Report upon Disease of the Heart, Derived from a Consideration of All the Cases Admitted into St. George’s Hospital during the Last Two Years and a Half

**Published:** 1851-09

**Authors:** 


*A Statistical Report upon Disease of the Heart, derived from a consideration of all the cases admitted into St. George's Hospital during the last two years and a half.* By Dr. Barclay.—Rheumatism is first considered as one of its causes. Divided into two nearly equal classes —those really inflammatory or acute, and those less so, or sub-acute, —the former class is found to contain sixty-seven cases with cardiac lesion, sixty-four without, and twenty-one doubtful. Endocardial murmur is found not to be certain evidence of disease, even in the most acute cases. Females are slightly more liable to acute rheumatism than males, but less liable to a recurrence of the disease. Females are more decidedly liable in a larger proportion to cardiac complication, and this is especially proved by the existence of friction-sound in the proportion of three females to two males. Cardiac complication exists eighteen or twenty per cent. more frequently in subsequent attacks than in primary ones. It is in the proportion of three to two of all the cases up to the age of twenty-five, and falls very rapidly after that age. The cases of sub-acute and chronic rheumatism furnish no examples of recent inflammation of the heart, but a considerable number of cases of old disease. So far as could be ascertained, these were almost all traceable to previous acute attacks, and were only about one-third of the cases which had previously suffered from acute rheumatism. The post-mortem appearances of recent inflammation are found associated with acute rheumatism, with disease of the kidney, with inflammation of the peritoneum and pleura, and with old disease of the heart, especially when hypertrophy existed, and with turbulent action during life. The cases of old disease of the heart are divided into sixty-one rheumatic, seventy non-rheumatic, and sixty-nine doubtful. They show a very considerable preponderance of males, especially among fatal cases. Up to the age of twenty, almost the whole, and even as far as thirty, more than half the cases, are associated with acute rheumatism. In the next twenty years, the non-rheumatic almost double the rheumatic cases, and after fifty, there are scarcely any derivable from rheumatism at all. The duration of rheumatic cases, dating from the first attack of acute rheumatism to death, is generally much longer for females than for males, varying in the latter from four to six years; in the former, from twelve to sixteen years. Four out of seven fatal cases of acute rheumatism, and twelve out of eighteen of older standing, are associated with pericarditis, which is always severe and extensive; but universal adhesion is neither the constant nor even the common result of rheumatic pericarditis, and it exists in cases where the previous existence of rheumatism is altogether denied. In valvular disease there are eighteen rheumatic cases, twenty-three non-rheumatic, and twelve doubtful The recent cases are all examples of inflammation of the mitral. When old and recent disease exist together, and when old disease is seen in different stages, the mitral valve generally appears to have been first attacked, and the aortic secondarily; and hence the preponderance of double valvular lesion in rheumatic cases, seems to be due to renewed inflammation at distinct periods. Inflammatory thickening occurs also in several cases in which there had been no rheumatism. Disease of the kidney is associated with two cases of simple recent fibrinous deposit on the valves, and three of recent pericarditis, in which no other cause was known to have been in operation. It seems questionable how far this can be taken as a cause of great thickening of the valves, or of an adherent pericardium. Disease of each set of valves seems to produce, in nearly equal proportions, hypertrophy and dilatation; but aortic regurgitation especially the latter, atheroma of the aorta, more commonly hypertrophy; adhesion of the pericardium, chiefly dilatation. Disease of the kidney is associated with an immense majority of the cases of hypertrophy, and similarly of the cases of disease of the kidney; more than a third presented on post-mortem examination more or less of hypertrophy of the heart. *Dublin Medical Press.*

